# Hazardous Removal of a Misplaced Nasogastric Tube

**DOI:** 10.5334/jbsr.2174

**Published:** 2020-08-05

**Authors:** Jean-Philippe Hardy, Benoît Ghaye, Francois Dermesropian

**Affiliations:** 1 Cliniques Universitaires Saint Luc, BE; 2 Grand Hopital de Charleroi, BE

**Keywords:** Pneumothorax, Naso-Gastric-Tube, Complication, chest x-ray, tubes

## Abstract

**Teaching point:** Careful analysis of tubes positioning on chest X-ray not only reveals misplacement but also helps to plan a safe removal.

## Case

**Figure 1:**
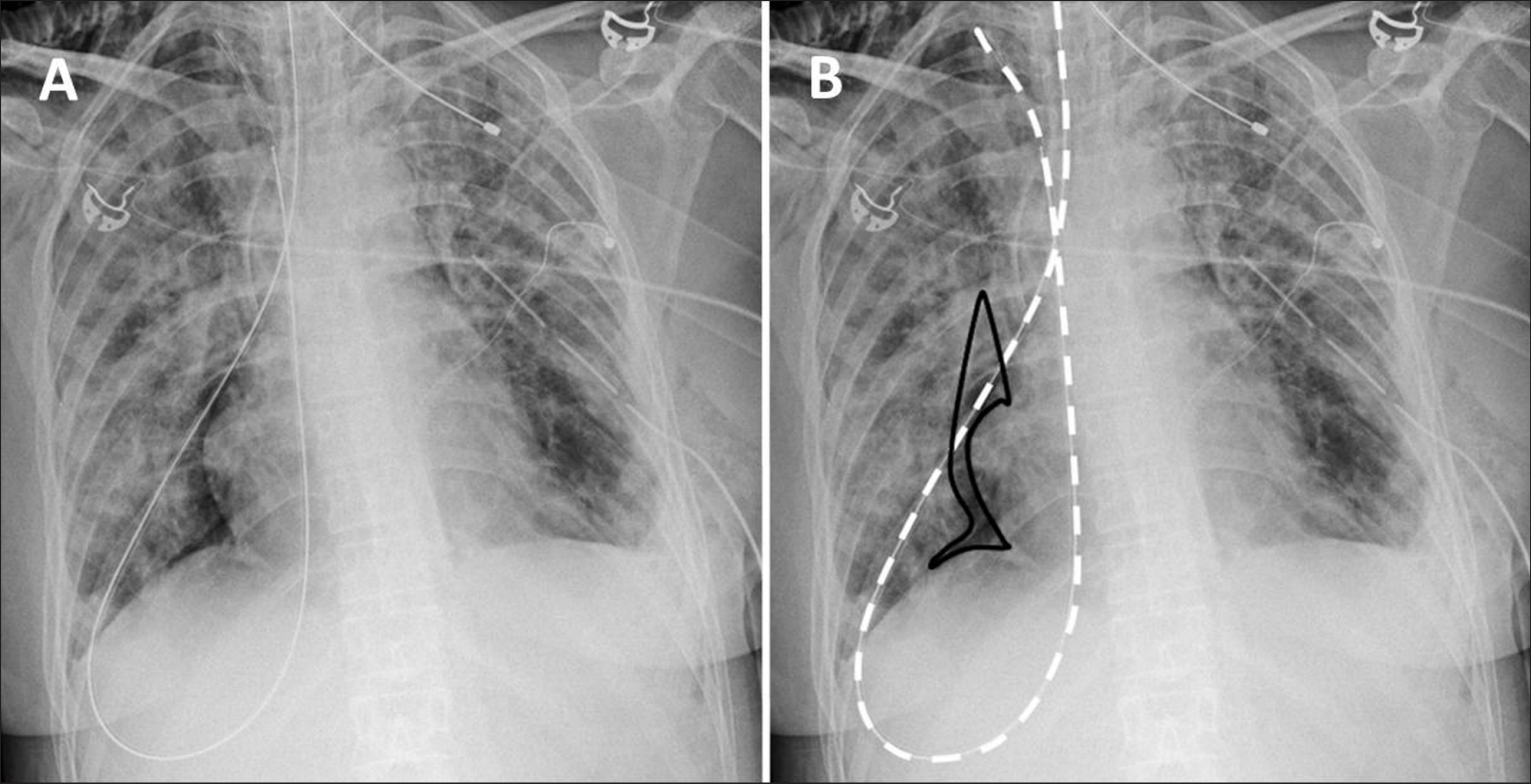


A 36-year-old woman was admitted to the intensive care unit for progressive dyspnea in a context of pulmonary fibrosis. Chest X-ray (Figure 1A) after insertion of a nasogastric tube (NGT) revealed a misplacement with the tube seemingly coursing downwards through the right main stem bronchus before looping in posterior pleural recess and heading toward the right pulmonary apex (Figure 1B, white dotted line). It also showed pneumothorax, manifesting as a right paracardiac radiolucency (black line). Shortly after removing this misplaced NGT, the patient went into cardiac arrest. Aware of the preexistent pneumothorax and in accordance to abnormal auscultation, tension pneumothorax was clinically suspected and thoracic drainage was performed, allowing resuscitation.

## Discussion

Many patients in intensive care units undergo insertion of NGT for indications including gastric decompression, aspiration prevention, administration of enteral nutrition or medication. Such a procedure is considered low-risk. However, misplacement of NGT into the airway occurs in 0.3% to 8% and may be associated with complications such as chemical pneumonia, pneumothorax, pulmonary hemorrhage, esophageal perforation, and intracranial placement. Because most of misplaced tubes are not associated with any sensation of resistance or cough, correct positioning must be evidenced before use to prevent those complications: auscultation and pH/bilirubin testing are bedside methods but chest X-ray remains the gold standard for determining correct NGT position [1].

This case emphasizes the high-risk procedure of removing that misplaced NGT tube: bronchopleural fistula and tension pneumothorax should be expected. Patient must require close monitoring following removal, and if needed, chest X-ray should be performed for early recognition and management of pneumothorax.
